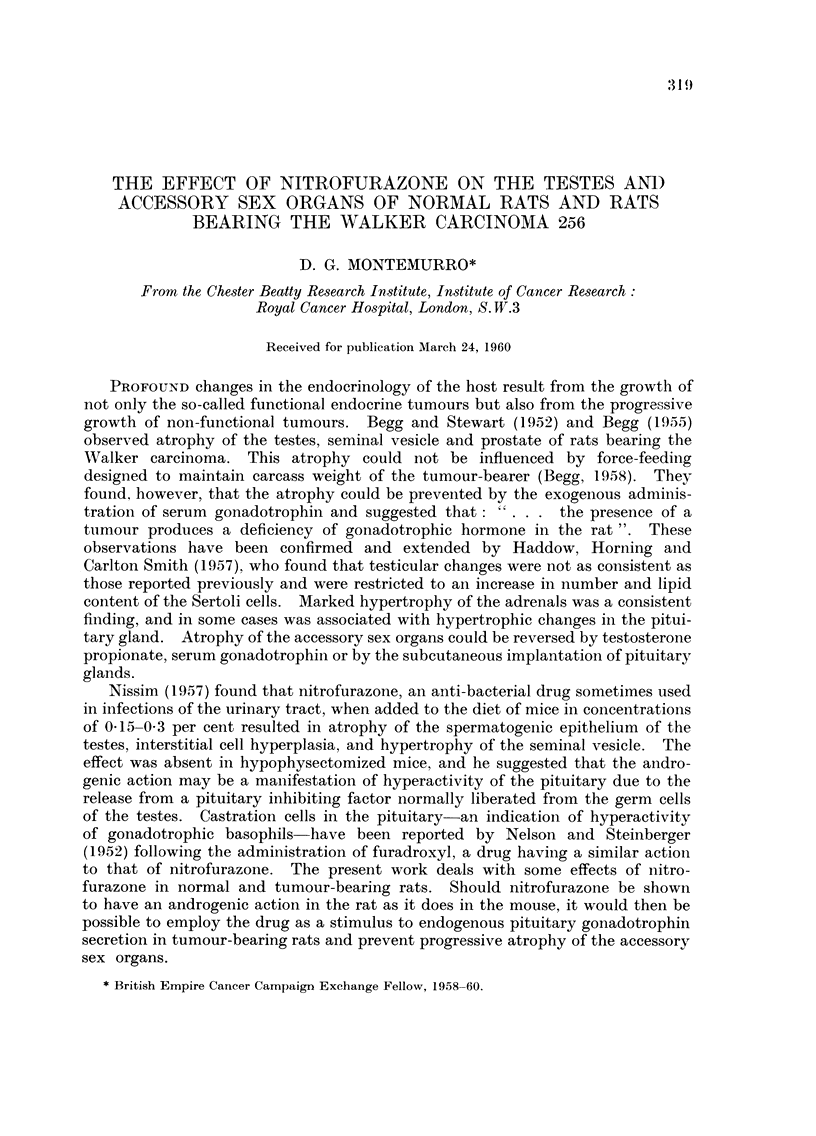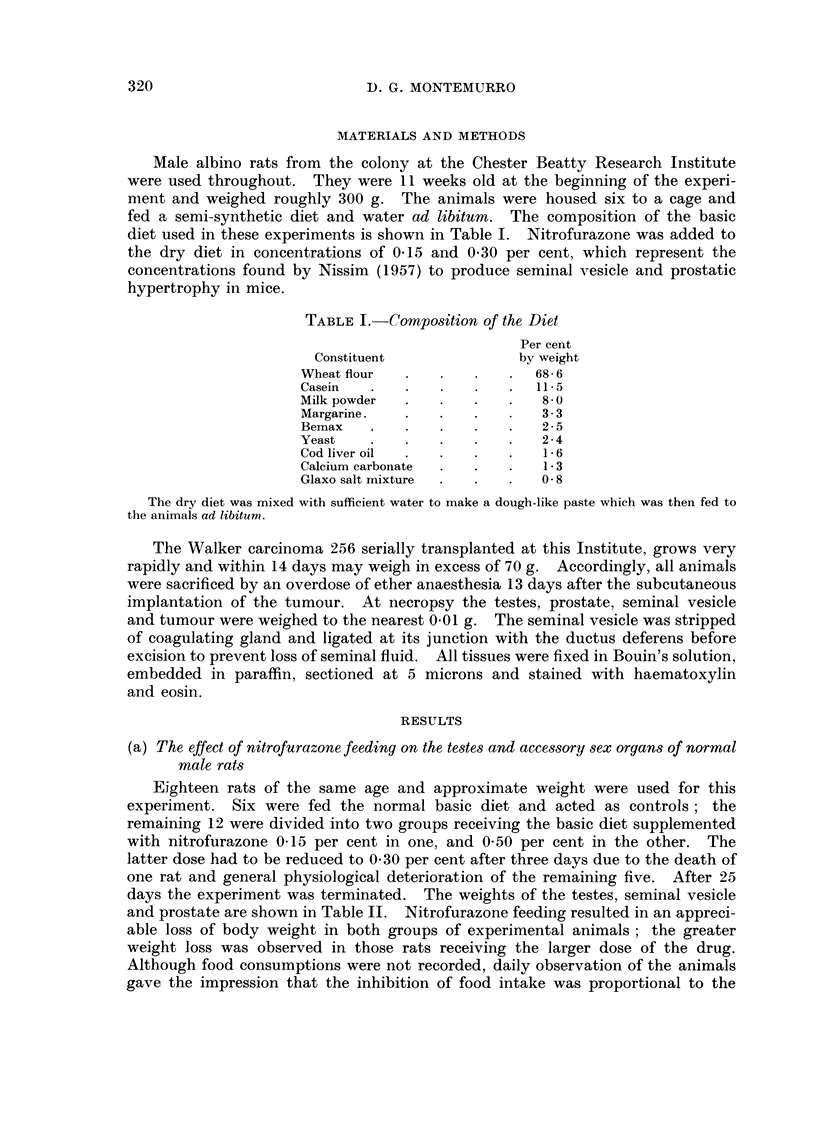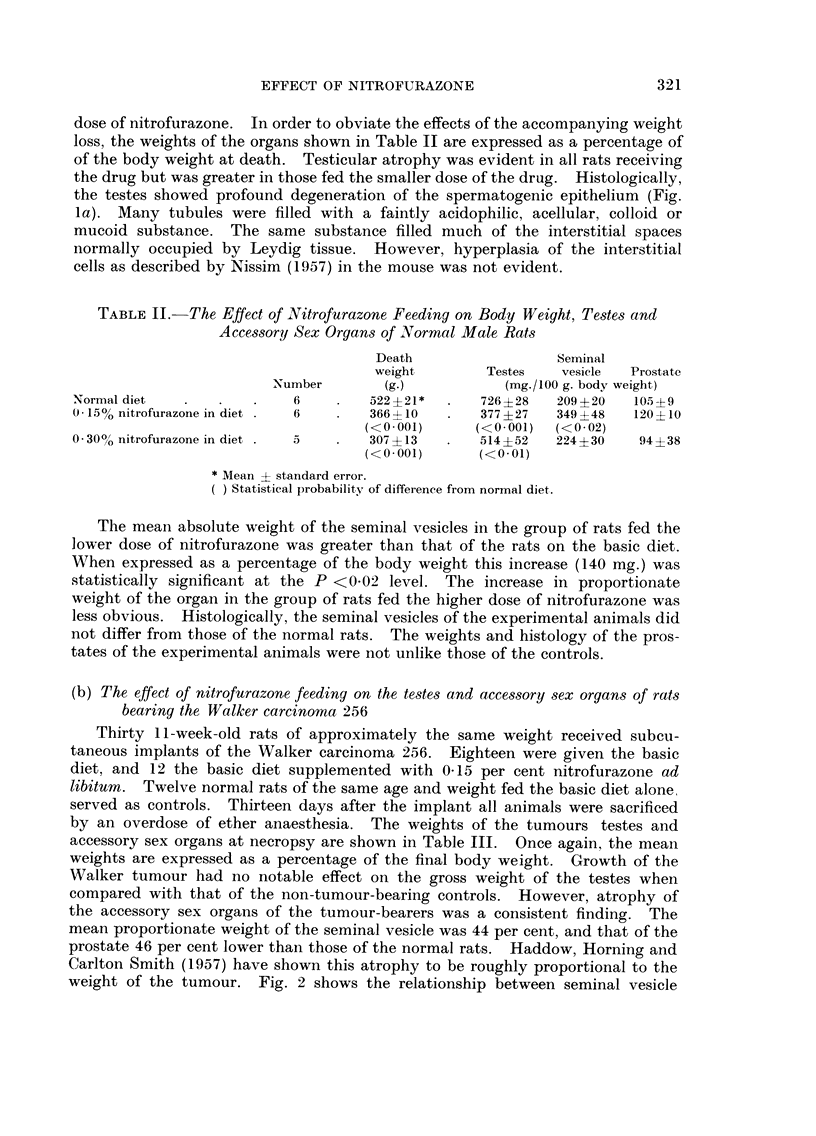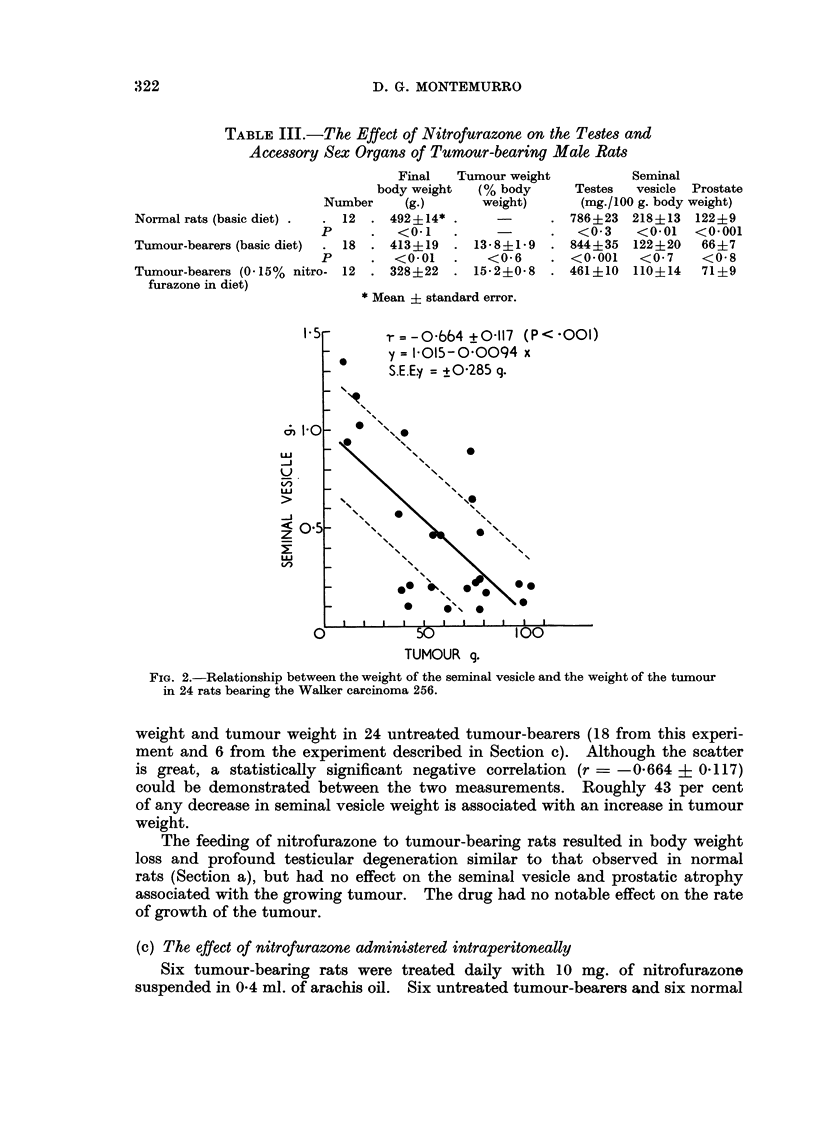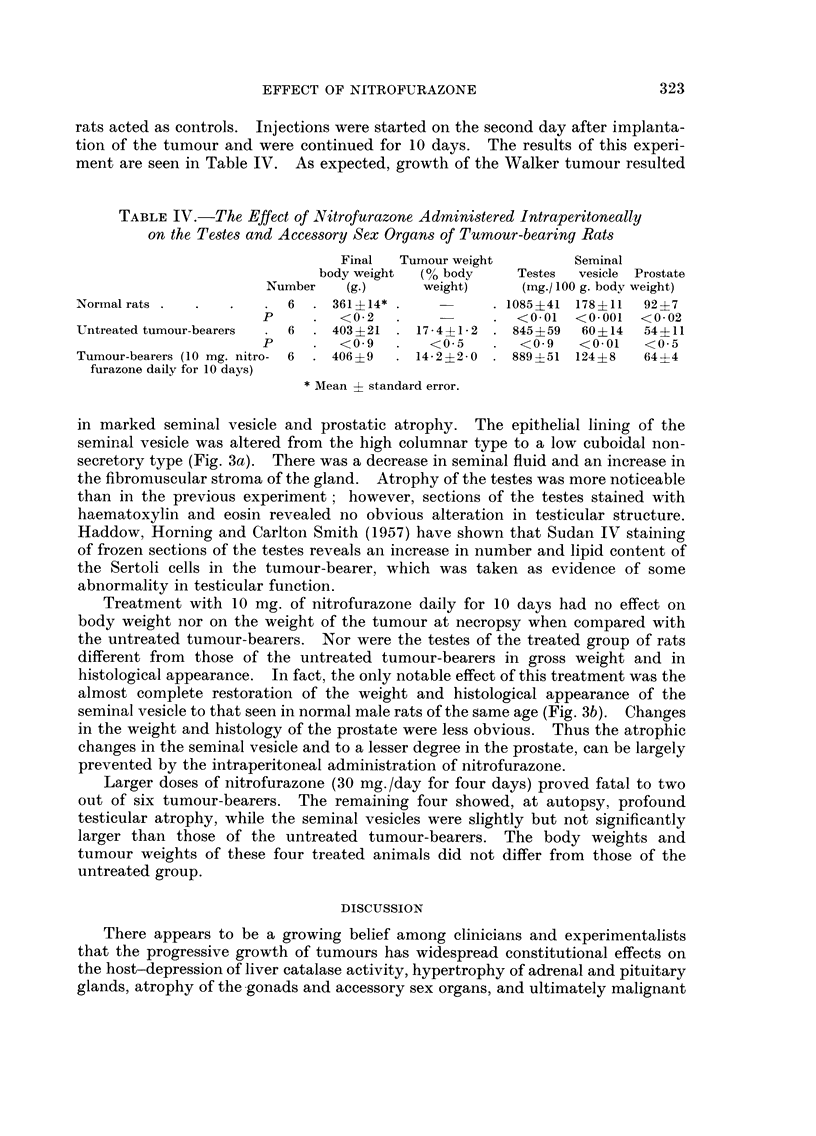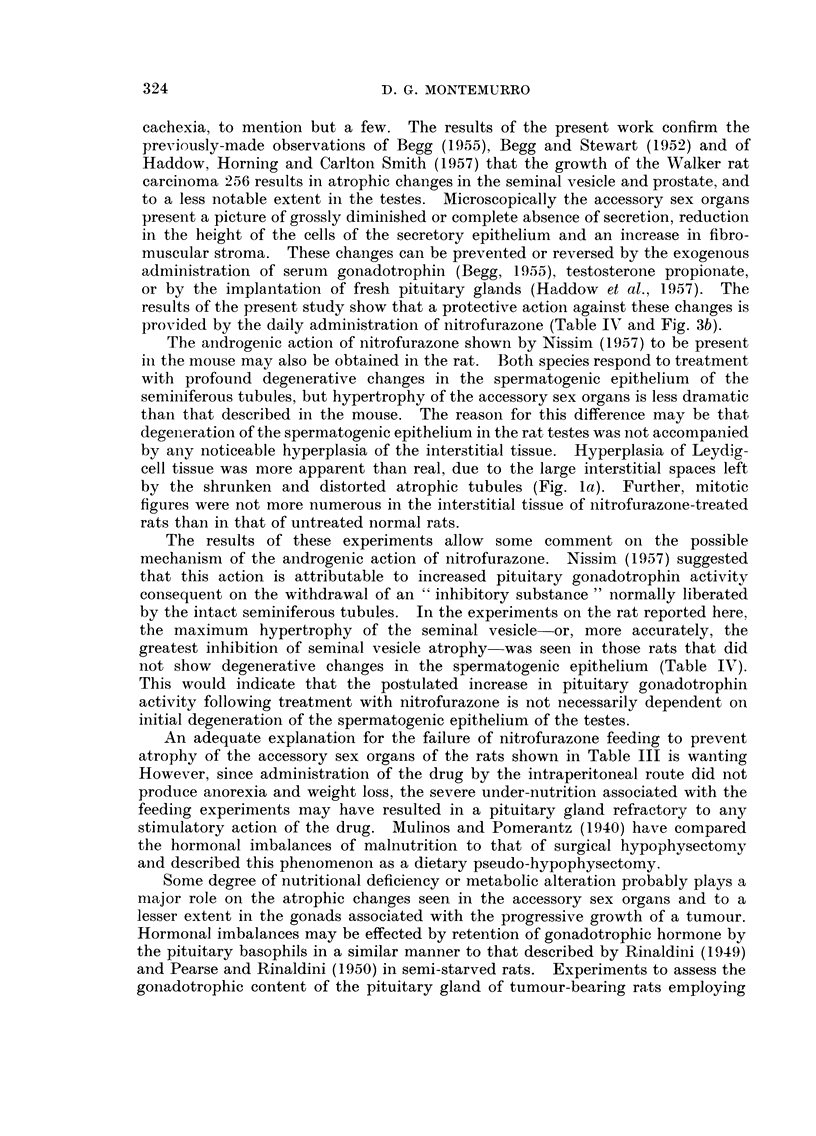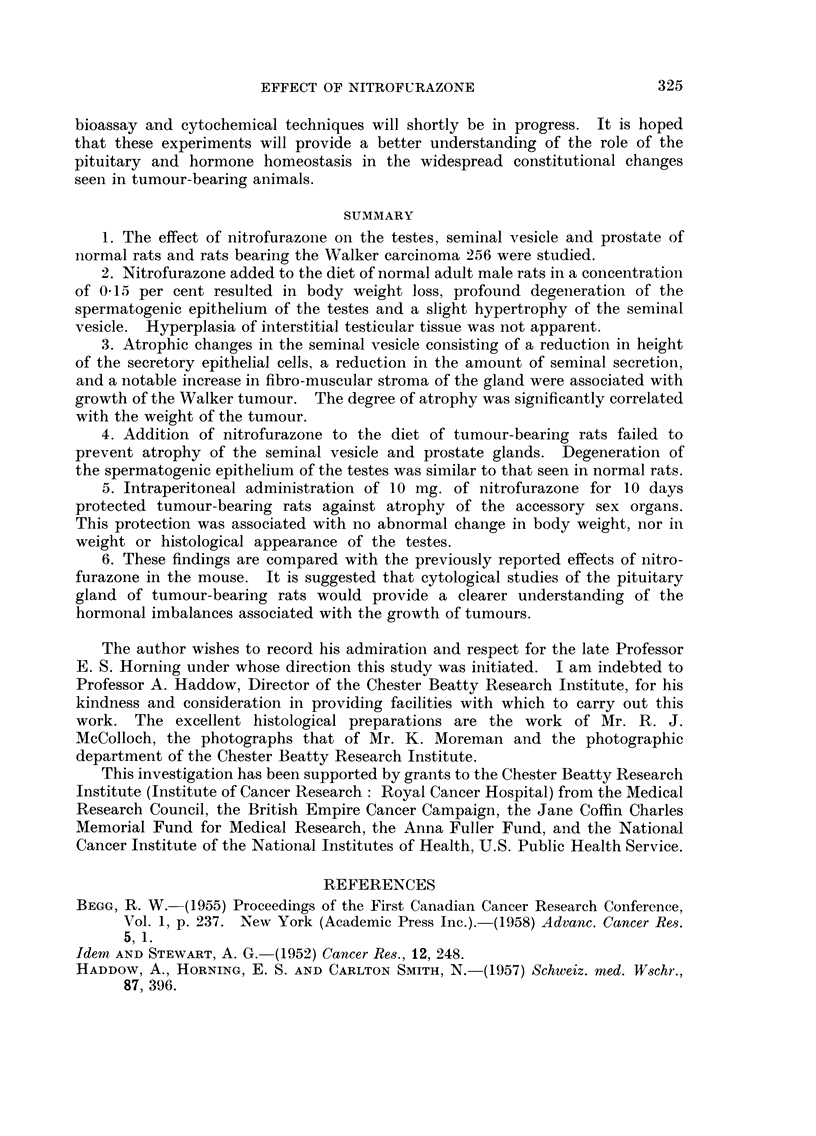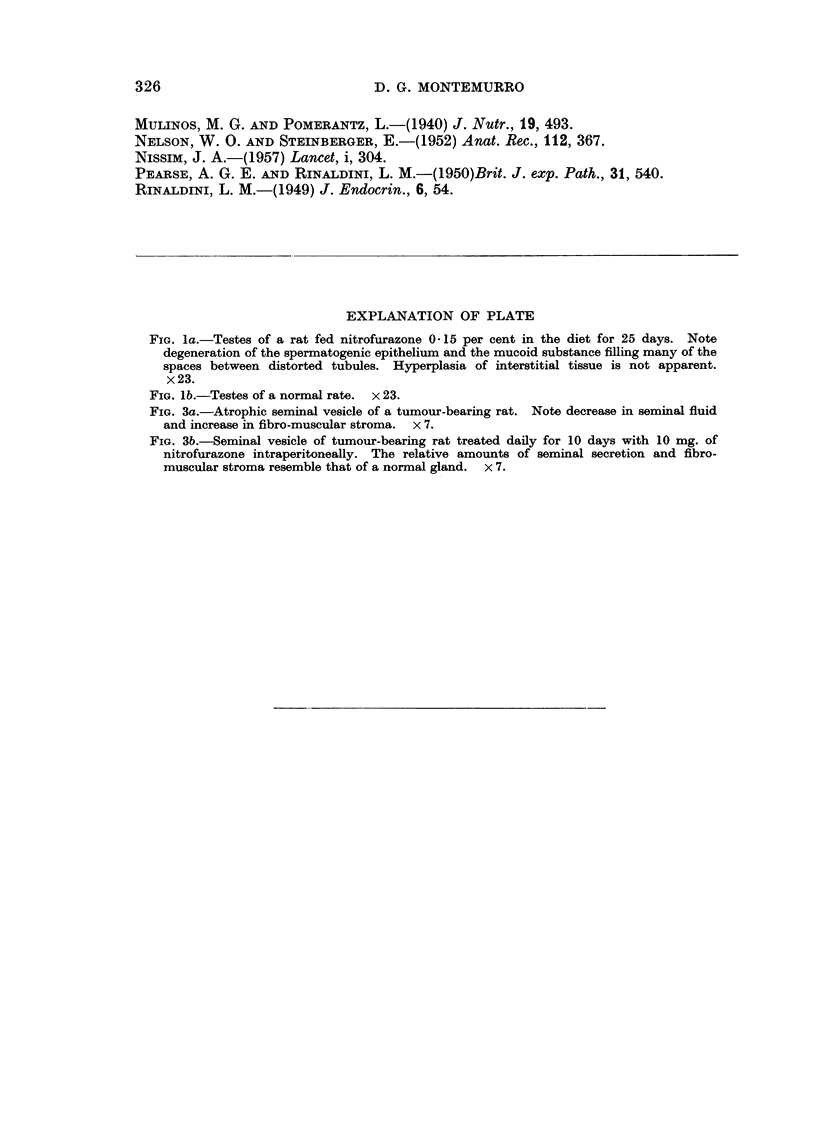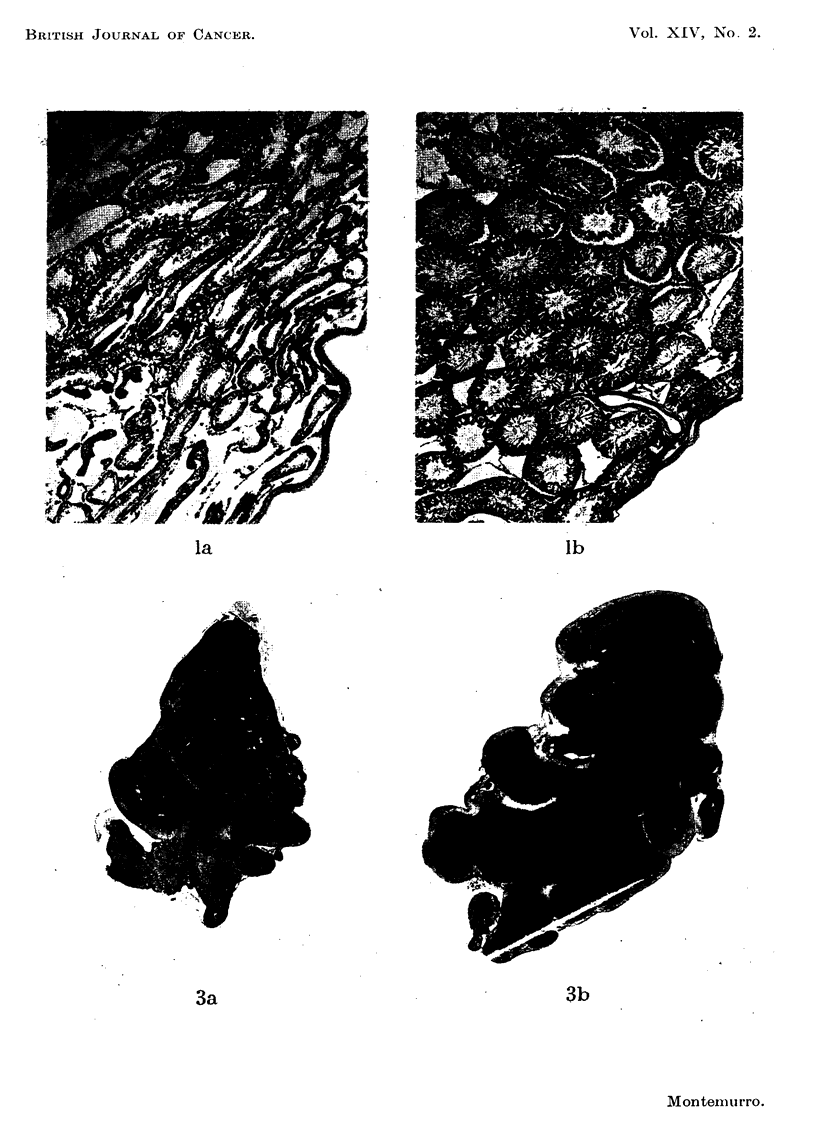# The Effect of Nitrofurazone on the Testes and Accessory Sex Organs of Normal Rats and Rats bearing the Walker Carcinoma 256

**DOI:** 10.1038/bjc.1960.38

**Published:** 1960-06

**Authors:** D. G. Montemurro

## Abstract

**Images:**


### THE EFFECT OF NITROFURAZONE ON THE TESTES AND ACCESSORY SEX ORGANS OF NORMAL RATS AND RATS BEARING THE WALKER CARCINOMA 256

D. G. MONTEMURRO*

*From the Chester Beatty Research Institute, Institute of Cancer Research : Royal Cancer Hospital, London, S.W.3*

Received for publication March 24, 1960

PROFOUND changes in the endocrinology of the host result from the growth of not only the so-called functional endocrine tumours but also from the progressive growth of non-functional tumours. Begg and Stewart (1952) and Begg (1955) observed atrophy of the testes, seminal vesicle and prostate of rats bearing the Walker carcinoma. This atrophy could not be influenced by force-feeding designed to maintain carcass weight of the tumour-bearer (Begg, 1958). They found, however, that the atrophy could be prevented by the exogenous administration of serum gonadotrophin and suggested that : " . . . the presence of a tumour produces a deficiency of gonadotrophic hormone in the rat ". These observations have been confirmed and extended by Haddow, Horning and Carlton Smith (1957), who found that testicular changes were not as consistent as those reported previously and were restricted to an increase in number and lipid content of the Sertoli cells. Marked hypertrophy of the adrenals was a consistent finding, and in some cases was associated with hypertrophic changes in the pituitary gland. Atrophy of the accessory sex organs could be reversed by testosterone propionate, serum gonadotrophin or by the subcutaneous implantation of pituitary glands.

Nissim (1957) found that nitrofurazone, an anti-bacterial drug sometimes used in infections of the urinary tract, when added to the diet of mice in concentrations of 0·15–0·3 per cent resulted in atrophy of the spermatogenic epithelium of the testes, interstitial cell hyperplasia, and hypertrophy of the seminal vesicle. The effect was absent in hypophysectomized mice, and he suggested that the androgenic action may be a manifestation of hyperactivity of the pituitary due to the release from a pituitary inhibiting factor normally liberated from the germ cells of the testes. Castration cells in the pituitary—an indication of hyperactivity of gonadotrophic basophils—have been reported by Nelson and Steinberger (1952) following the administration of furadroxyl, a drug having a similar action to that of nitrofurazone. The present work deals with some effects of nitrofurazone in normal and tumour-bearing rats. Should nitrofurazone be shown to have an androgenic action in the rat as it does in the mouse, it would then be possible to employ the drug as a stimulus to endogenous pituitary gonadotrophin secretion in tumour-bearing rats and prevent progressive atrophy of the accessory sex organs.

* British Empire Cancer Campaign Exchange Fellow, 1958–60.

### MATERIALS AND METHODS

Male albino rats from the colony at the Chester Beatty Research Institute were used throughout. They were 11 weeks old at the beginning of the experiment and weighed roughly 300 g. The animals were housed six to a cage and fed a semi-synthetic diet and water *ad libitum.* The composition of the basic diet used in these experiments is shown in Table I. Nitrofurazone was added to the dry diet in concentrations of 0·15 and 0·30 per cent, which represent the concentrations found by Nissim (1957) to produce seminal vesicle and prostatic hypertrophy in mice.

TABLE I.—*Composition of the Diet*

| Constituent | Per cent by weight |
|---|---|
| Wheat flour | 68·6 |
| Casein | 11·5 |
| Milk powder | 8·0 |
| Margarine | 3·3 |
| Bemax | 2·5 |
| Yeast | 2·4 |
| Cod liver oil | 1·6 |
| Calcium carbonate | 1·3 |
| Glaxo salt mixture | 0·8 |

The dry diet was mixed with sufficient water to make a dough-like paste which was then fed to the animals *ad libitum.*

The Walker carcinoma 256 serially transplanted at this Institute, grows very rapidly and within 14 days may weigh in excess of 70 g. Accordingly, all animals were sacrificed by an overdose of ether anaesthesia 13 days after the subcutaneous implantation of the tumour. At necropsy the testes, prostate, seminal vesicle and tumour were weighed to the nearest 0·01 g. The seminal vesicle was stripped of coagulating gland and ligated at its junction with the ductus deferens before excision to prevent loss of seminal fluid. All tissues were fixed in Bouin's solution, embedded in paraffin, sectioned at 5 microns and stained with haematoxylin and eosin.

### RESULTS

### (a) *The effect of nitrofurazone feeding on the testes and accessory sex organs of normal male rats*

Eighteen rats of the same age and approximate weight were used for this experiment. Six were fed the normal basic diet and acted as controls ; the remaining 12 were divided into two groups receiving the basic diet supplemented with nitrofurazone 0·15 per cent in one, and 0·50 per cent in the other. The latter dose had to be reduced to 0·30 per cent after three days due to the death of one rat and general physiological deterioration of the remaining five. After 25 days the experiment was terminated. The weights of the testes, seminal vesicle and prostate are shown in Table II. Nitrofurazone feeding resulted in an appreciable loss of body weight in both groups of experimental animals ; the greater weight loss was observed in those rats receiving the larger dose of the drug. Although food consumptions were not recorded, daily observation of the animals gave the impression that the inhibition of food intake was proportional to the dose of nitrofurazone. In order to obviate the effects of the accompanying weight loss, the weights of the organs shown in Table II are expressed as a percentage of of the body weight at death. Testicular atrophy was evident in all rats receiving the drug but was greater in those fed the smaller dose of the drug. Histologically, the testes showed profound degeneration of the spermatogenic epithelium (Fig. 1*a*). Many tubules were filled with a faintly acidophilic, acellular, colloid or mucoid substance. The same substance filled much of the interstitial spaces normally occupied by Leydig tissue. However, hyperplasia of the interstitial cells as described by Nissim (1957) in the mouse was not evident.

TABLE II.—*The Effect of Nitrofurazone Feeding on Body Weight, Testes and Accessory Sex Organs of Normal Male Rats*

| | Number | Death weight (g.) | Testes | Seminal vesicle | Prostate |
|---|---|---|---|---|---|
| | | | (mg./100 g. body weight) | | |
| Normal diet | 6 | 522±21* | 726±28 | 209±20 | 105±9 |
| 0·15% nitrofurazone in diet | 6 | 366±10 (<0·001) | 377±27 (<0·001) | 349±48 (<0·02) | 120±10 |
| 0·30% nitrofurazone in diet | 5 | 307±13 (<0·001) | 514±52 (<0·01) | 224±30 | 94±38 |

* Mean ± standard error.
( ) Statistical probability of difference from normal diet.

The mean absolute weight of the seminal vesicles in the group of rats fed the lower dose of nitrofurazone was greater than that of the rats on the basic diet. When expressed as a percentage of the body weight this increase (140 mg.) was statistically significant at the $P < 0{\cdot}02$ level. The increase in proportionate weight of the organ in the group of rats fed the higher dose of nitrofurazone was less obvious. Histologically, the seminal vesicles of the experimental animals did not differ from those of the normal rats. The weights and histology of the prostates of the experimental animals were not unlike those of the controls.

(b) *The effect of nitrofurazone feeding on the testes and accessory sex organs of rats bearing the Walker carcinoma* 256

Thirty 11-week-old rats of approximately the same weight received subcutaneous implants of the Walker carcinoma 256. Eighteen were given the basic diet, and 12 the basic diet supplemented with 0·15 per cent nitrofurazone *ad libitum*. Twelve normal rats of the same age and weight fed the basic diet alone, served as controls. Thirteen days after the implant all animals were sacrificed by an overdose of ether anaesthesia. The weights of the tumours testes and accessory sex organs at necropsy are shown in Table III. Once again, the mean weights are expressed as a percentage of the final body weight. Growth of the Walker tumour had no notable effect on the gross weight of the testes when compared with that of the non-tumour-bearing controls. However, atrophy of the accessory sex organs of the tumour-bearers was a consistent finding. The mean proportionate weight of the seminal vesicle was 44 per cent, and that of the prostate 46 per cent lower than those of the normal rats. Haddow, Horning and Carlton Smith (1957) have shown this atrophy to be roughly proportional to the weight of the tumour. Fig. 2 shows the relationship between seminal vesicle

TABLE III.—*The Effect of Nitrofurazone on the Testes and Accessory Sex Organs of Tumour-bearing Male Rats*

| | Number | Final body weight (g.) | Tumour weight (% body weight) | Testes (mg./100 g. body weight) | Seminal vesicle (mg./100 g. body weight) | Prostate (mg./100 g. body weight) |
|---|---|---|---|---|---|---|
| Normal rats (basic diet) | 12 | 492±14* | — | 786±23 | 218±13 | 122±9 |
| *P* | | <0·1 | — | <0·3 | <0·01 | <0·001 |
| Tumour-bearers (basic diet) | 18 | 413±19 | 13·8±1·9 | 844±35 | 122±20 | 66±7 |
| *P* | | <0·01 | <0·6 | <0·001 | <0·7 | <0·8 |
| Tumour-bearers (0·15% nitrofurazone in diet) | 12 | 328±22 | 15·2±0·8 | 461±10 | 110±14 | 71±9 |

* Mean ± standard error.

FIG. 2.—Relationship between the weight of the seminal vesicle and the weight of the tumour in 24 rats bearing the Walker carcinoma 256.

weight and tumour weight in 24 untreated tumour-bearers (18 from this experiment and 6 from the experiment described in Section c). Although the scatter is great, a statistically significant negative correlation ($r = -0{\cdot}664 \pm 0{\cdot}117$) could be demonstrated between the two measurements. Roughly 43 per cent of any decrease in seminal vesicle weight is associated with an increase in tumour weight.

The feeding of nitrofurazone to tumour-bearing rats resulted in body weight loss and profound testicular degeneration similar to that observed in normal rats (Section a), but had no effect on the seminal vesicle and prostatic atrophy associated with the growing tumour. The drug had no notable effect on the rate of growth of the tumour.

(c) *The effect of nitrofurazone administered intraperitoneally*

Six tumour-bearing rats were treated daily with 10 mg. of nitrofurazone suspended in 0·4 ml. of arachis oil. Six untreated tumour-bearers and six normal rats acted as controls. Injections were started on the second day after implantation of the tumour and were continued for 10 days. The results of this experiment are seen in Table IV. As expected, growth of the Walker tumour resulted

TABLE IV.—*The Effect of Nitrofurazone Administered Intraperitoneally on the Testes and Accessory Sex Organs of Tumour-bearing Rats*

| | Number | Final body weight (g.) | Tumour weight (% body weight) | Testes (mg./100 g. body weight) | Seminal vesicle (mg./100 g. body weight) | Prostate (mg./100 g. body weight) |
|---|---|---|---|---|---|---|
| Normal rats | 6 | 361±14* | — | 1085±41 | 178±11 | 92±7 |
| *P* | | <0·2 | — | <0·01 | <0·001 | <0·02 |
| Untreated tumour-bearers | 6 | 403±21 | 17·4±1·2 | 845±59 | 60±14 | 54±11 |
| *P* | | <0·9 | <0·5 | <0·9 | <0·01 | <0·5 |
| Tumour-bearers (10 mg. nitrofurazone daily for 10 days) | 6 | 406±9 | 14·2±2·0 | 889±51 | 124±8 | 64±4 |

* Mean ± standard error.

in marked seminal vesicle and prostatic atrophy. The epithelial lining of the seminal vesicle was altered from the high columnar type to a low cuboidal non-secretory type (Fig. 3*a*). There was a decrease in seminal fluid and an increase in the fibromuscular stroma of the gland. Atrophy of the testes was more noticeable than in the previous experiment ; however, sections of the testes stained with haematoxylin and eosin revealed no obvious alteration in testicular structure. Haddow, Horning and Carlton Smith (1957) have shown that Sudan IV staining of frozen sections of the testes reveals an increase in number and lipid content of the Sertoli cells in the tumour-bearer, which was taken as evidence of some abnormality in testicular function.

Treatment with 10 mg. of nitrofurazone daily for 10 days had no effect on body weight nor on the weight of the tumour at necropsy when compared with the untreated tumour-bearers. Nor were the testes of the treated group of rats different from those of the untreated tumour-bearers in gross weight and in histological appearance. In fact, the only notable effect of this treatment was the almost complete restoration of the weight and histological appearance of the seminal vesicle to that seen in normal male rats of the same age (Fig. 3*b*). Changes in the weight and histology of the prostate were less obvious. Thus the atrophic changes in the seminal vesicle and to a lesser degree in the prostate, can be largely prevented by the intraperitoneal administration of nitrofurazone.

Larger doses of nitrofurazone (30 mg./day for four days) proved fatal to two out of six tumour-bearers. The remaining four showed, at autopsy, profound testicular atrophy, while the seminal vesicles were slightly but not significantly larger than those of the untreated tumour-bearers. The body weights and tumour weights of these four treated animals did not differ from those of the untreated group.

### DISCUSSION

There appears to be a growing belief among clinicians and experimentalists that the progressive growth of tumours has widespread constitutional effects on the host–depression of liver catalase activity, hypertrophy of adrenal and pituitary glands, atrophy of the gonads and accessory sex organs, and ultimately malignant cachexia, to mention but a few. The results of the present work confirm the previously-made observations of Begg (1955), Begg and Stewart (1952) and of Haddow, Horning and Carlton Smith (1957) that the growth of the Walker rat carcinoma 256 results in atrophic changes in the seminal vesicle and prostate, and to a less notable extent in the testes. Microscopically the accessory sex organs present a picture of grossly diminished or complete absence of secretion, reduction in the height of the cells of the secretory epithelium and an increase in fibromuscular stroma. These changes can be prevented or reversed by the exogenous administration of serum gonadotrophin (Begg, 1955), testosterone propionate, or by the implantation of fresh pituitary glands (Haddow *et al.*, 1957). The results of the present study show that a protective action against these changes is provided by the daily administration of nitrofurazone (Table IV and Fig. 3*b*).

The androgenic action of nitrofurazone shown by Nissim (1957) to be present in the mouse may also be obtained in the rat. Both species respond to treatment with profound degenerative changes in the spermatogenic epithelium of the seminiferous tubules, but hypertrophy of the accessory sex organs is less dramatic than that described in the mouse. The reason for this difference may be that degeneration of the spermatogenic epithelium in the rat testes was not accompanied by any noticeable hyperplasia of the interstitial tissue. Hyperplasia of Leydig-cell tissue was more apparent than real, due to the large interstitial spaces left by the shrunken and distorted atrophic tubules (Fig. 1*a*). Further, mitotic figures were not more numerous in the interstitial tissue of nitrofurazone-treated rats than in that of untreated normal rats.

The results of these experiments allow some comment on the possible mechanism of the androgenic action of nitrofurazone. Nissim (1957) suggested that this action is attributable to increased pituitary gonadotrophin activity consequent on the withdrawal of an " inhibitory substance " normally liberated by the intact seminiferous tubules. In the experiments on the rat reported here, the maximum hypertrophy of the seminal vesicle—or, more accurately, the greatest inhibition of seminal vesicle atrophy—was seen in those rats that did not show degenerative changes in the spermatogenic epithelium (Table IV). This would indicate that the postulated increase in pituitary gonadotrophin activity following treatment with nitrofurazone is not necessarily dependent on initial degeneration of the spermatogenic epithelium of the testes.

An adequate explanation for the failure of nitrofurazone feeding to prevent atrophy of the accessory sex organs of the rats shown in Table III is wanting However, since administration of the drug by the intraperitoneal route did not produce anorexia and weight loss, the severe under-nutrition associated with the feeding experiments may have resulted in a pituitary gland refractory to any stimulatory action of the drug. Mulinos and Pomerantz (1940) have compared the hormonal imbalances of malnutrition to that of surgical hypophysectomy and described this phenomenon as a dietary pseudo-hypophysectomy.

Some degree of nutritional deficiency or metabolic alteration probably plays a major role on the atrophic changes seen in the accessory sex organs and to a lesser extent in the gonads associated with the progressive growth of a tumour. Hormonal imbalances may be effected by retention of gonadotrophic hormone by the pituitary basophils in a similar manner to that described by Rinaldini (1949) and Pearse and Rinaldini (1950) in semi-starved rats. Experiments to assess the gonadotrophic content of the pituitary gland of tumour-bearing rats employing bioassay and cytochemical techniques will shortly be in progress. It is hoped that these experiments will provide a better understanding of the role of the pituitary and hormone homeostasis in the widespread constitutional changes seen in tumour-bearing animals.

SUMMARY

1. The effect of nitrofurazone on the testes, seminal vesicle and prostate of normal rats and rats bearing the Walker carcinoma 256 were studied.

2. Nitrofurazone added to the diet of normal adult male rats in a concentration of 0·15 per cent resulted in body weight loss, profound degeneration of the spermatogenic epithelium of the testes and a slight hypertrophy of the seminal vesicle. Hyperplasia of interstitial testicular tissue was not apparent.

3. Atrophic changes in the seminal vesicle consisting of a reduction in height of the secretory epithelial cells, a reduction in the amount of seminal secretion, and a notable increase in fibro-muscular stroma of the gland were associated with growth of the Walker tumour. The degree of atrophy was significantly correlated with the weight of the tumour.

4. Addition of nitrofurazone to the diet of tumour-bearing rats failed to prevent atrophy of the seminal vesicle and prostate glands. Degeneration of the spermatogenic epithelium of the testes was similar to that seen in normal rats.

5. Intraperitoneal administration of 10 mg. of nitrofurazone for 10 days protected tumour-bearing rats against atrophy of the accessory sex organs. This protection was associated with no abnormal change in body weight, nor in weight or histological appearance of the testes.

6. These findings are compared with the previously reported effects of nitrofurazone in the mouse. It is suggested that cytological studies of the pituitary gland of tumour-bearing rats would provide a clearer understanding of the hormonal imbalances associated with the growth of tumours.

The author wishes to record his admiration and respect for the late Professor E. S. Horning under whose direction this study was initiated. I am indebted to Professor A. Haddow, Director of the Chester Beatty Research Institute, for his kindness and consideration in providing facilities with which to carry out this work. The excellent histological preparations are the work of Mr. R. J. McColloch, the photographs that of Mr. K. Moreman and the photographic department of the Chester Beatty Research Institute.

This investigation has been supported by grants to the Chester Beatty Research Institute (Institute of Cancer Research : Royal Cancer Hospital) from the Medical Research Council, the British Empire Cancer Campaign, the Jane Coffin Charles Memorial Fund for Medical Research, the Anna Fuller Fund, and the National Cancer Institute of the National Institutes of Health, U.S. Public Health Service.

### REFERENCES

BEGG, R. W.—(1955) Proceedings of the First Canadian Cancer Research Conference, Vol. 1, p. 237. New York (Academic Press Inc.).—(1958) *Advanc. Cancer Res.* **5**, 1.

*Idem* AND STEWART, A. G.—(1952) *Cancer Res.*, **12**, 248.

HADDOW, A., HORNING, E. S. AND CARLTON SMITH, N.—(1957) *Schweiz. med. Wschr.*, **87**, 396.

MULINOS, M. G. AND POMERANTZ, L.—(1940) *J. Nutr.*, **19**, 493.
NELSON, W. O. AND STEINBERGER, E.—(1952) *Anat. Rec.*, **112**, 367.
NISSIM, J. A.—(1957) *Lancet*, i, 304.
PEARSE, A. G. E. AND RINALDINI, L. M.—(1950)*Brit. J. exp. Path.*, **31**, 540.
RINALDINI, L. M.—(1949) *J. Endocrin.*, **6**, 54.

---

### EXPLANATION OF PLATE

FIG. 1*a*.—Testes of a rat fed nitrofurazone 0·15 per cent in the diet for 25 days. Note degeneration of the spermatogenic epithelium and the mucoid substance filling many of the spaces between distorted tubules. Hyperplasia of interstitial tissue is not apparent. ×23.

FIG. 1*b*.—Testes of a normal rate. ×23.

FIG. 3*a*.—Atrophic seminal vesicle of a tumour-bearing rat. Note decrease in seminal fluid and increase in fibro-muscular stroma. ×7.

FIG. 3*b*.—Seminal vesicle of tumour-bearing rat treated daily for 10 days with 10 mg. of nitrofurazone intraperitoneally. The relative amounts of seminal secretion and fibro-muscular stroma resemble that of a normal gland. ×7.